# “When I Retire, I’ll Move Out of the City”: Mental Well-being of the Elderly in Rural vs. Urban Settings

**DOI:** 10.3390/ijerph17072442

**Published:** 2020-04-03

**Authors:** Manuela Alcañiz, Maria-Carme Riera-Prunera, Aïda Solé-Auró

**Affiliations:** 1 Riskcenter, Department of Econometrics, Statistics and Applied Economy, Universitat de Barcelona, 08034 Barcelona, Spain; 2 AQR Research Group, Department of Econometrics, Statistics and Applied Economy, Universitat de Barcelona, 08034 Barcelona, Spain; mcriera-prunera@ub.edu; 3 DemoSoc Research Group, Department of Political and Social Sciences, Universitat Pompeu Fabra, 08005 Barcelona, Spain; aida.sole@upf.edu

**Keywords:** mental well-being, rurality, longevity

## Abstract

This study examines the influence of risk factors on mental well-being at older ages focusing on the level of rurality of the living environment. We used cross-sectional, nationally representative survey data for Catalonia (Spain) from 2015 to 2017 to explain the mental well-being of the population aged 65 years and over. Based on a sample of 2621 individuals, we created a score of current mental well-being using the Short Warwick-Edinburgh Mental Well-being Scale (SWEMWBS). Using logistic regression and non-parametric tests, we identified the sociodemographic, health and lifestyle variables which, in combination with the features of the rural and urban settings of the living space, were associated with poor SWEMWBS scores. Our results reveal that adequate social support is linked to expectations of good mental well-being in later life. Poor self-perceived health and ageing limitations are associated with less deterioration of the well-being for the elderly living in rural areas, whereas living in urban areas is linked to a higher risk of suffering from emotional distress attributable to economic difficulties or low educational attainment. Incentivizing older people to live in rural environments could result in greater well-being in the last stages of life; appropriate prospective studies are needed to test this positive outcome.

## 1. Introduction

European life expectancy has undergone a rapid increase over the last two centuries, accompanied by an enormous growth in population. The proportion of the world’s population aged over 60 is set to almost double between 2015 and 2050, rising from 12 to 22% [1]. These figures represent a serious challenge for society, which will have to ensure successful ageing for a significant amount of the population. Concern for quality of life in advanced ages also means taking care of the mental well-being of the elderly. In this respect, special attention needs to be paid to those living in rural areas, as their emotional stability might be conditioned by subjective perceptions of abandonment or isolation, a lack of social support or reduced social networks [2].

### 1.1. Mental Well-being among Older Individuals

The mental well-being of older adults refers to how the elderly perceive their day-to-day existence, that is, whether their outlook is positive or negative, which, in turn, determines just how pleasant or unpleasant life can be. A positive sense of emotional well-being enables individuals to function effectively and feel integrated in society; moreover, those with good mental health have an increased ability to recover effectively from illness, change or misfortune [3].

Depressive symptoms in advanced ages appear to account for associated patterns of mental well-being. Regardless of age, Western populations were found to have higher levels of depression than Asian populations [4]. Some studies claim that the prevalence of depressive symptoms is higher in urban areas [5,6], as residents are exposed to greater levels of stress related to housing, work, marriage, childbearing and insecurity, together with the concentration of poverty in certain city areas and poor social integration. Chronic disease is also a major factor influencing well-being in older adults. Such diseases are associated with experiencing less enjoyment when completing day-to-day activities [7]. The better an individual’s health status, the more positive is their well-being [8]. This means that successful ageing at very old ages begins in the younger stages of the life cycle [9].

Another key element for ensuring mental well-being is social support [10]. Such support is an essential determinant of an elderly person’s health and functioning in their living environment [11,12]. Engagement in social activities and caring for grandchildren are additional elements that can be particularly beneficial for maintaining older people’s health and subjective well-being [13,14]. Gender may also play a role: greater social participation had protective effects on depressive symptoms for women [15]. Similarly, females were more likely to report depressive symptoms, related to sex differences in the perception of emotions [16] or to women’s living longer and, thus, suffering more chronic diseases and experiencing a greater loss of relatives and friends [17].

Levels of education and income may also influence mental well-being. Some results for Europe show that educational inequalities in health can be partially explained by different levels of active ageing engagement [18], as older people with high levels of education reported higher engagement in active ageing activities. Evidence from the US [19] found that income and education are closely related to evaluation of life, while health, care giving, loneliness and smoking are more closely related to daily emotions. The same authors conclude that high incomes can buy life satisfaction but not happiness, and that low incomes are associated with low life evaluation and low emotional well-being. Mixed evidence is also reported, pointing out that social relationships and aspects of the social environment have the potential for both health promoting and health damaging effects in older adults [20].

### 1.2. Association between Living Space and Mental Well-being

There are various definitions of “rural” settings, each emphasizing different criteria, for example, population size and density, or environmental context. The “rural and small town” definition, i.e., the population living in a municipality outside the commuting zone of a larger urban center, has been recommended as a starting point [21]. Cities and densely populated areas tend to be associated with noise, agitation, stress, and anxiety [22] and people living there are at greater risk of suffering from poor mental health [23]. In contrast, rural areas can be expected to be quiet and peaceful, which has been identified as a reason for adults nearing retirement to express their desire to move out of the city [24].

However, while some studies suggest that the rural environment can have a positive effect on mental well-being [25], especially in developed environments [26,27], others emphasize the negative impact of living isolated in rural communities [28], and the stigma residents may be subject to when seeking help to treat their mental health problems, given that communities are small and social networks do not favor privacy [29]. Psychosocial factors are also a more important determinant of affective or anxiety disorders than the isolation of communities per se [30]. Eastern European countries outside the European Union (EU) seem to be more diverse in terms of the well-being of their older people than are nearby EU member states [31], while frequent offspring contact is much more unlikely among northern Europeans than among their southern counterparts [32]. Moreover, older northern Europeans prefer to live alone, partly as they seek greater autonomy and independence.

Across the continent, much of rural Europe has witnessed vast changes over the last two decades, including major demographic and economic shifts that have had an impact on the residents’ quality of life [33]. Spain is no exception in this regard and, despite the obvious importance of the effect of the living environment on mental well-being, this issue has not been examined with sufficient depth as it impacts older adults. It is our contention that the level of rurality in the area in which older people live can have an effect on their subjective life evaluations. To shed light on this relationship, we conducted a study in Catalonia, a Mediterranean region in the northeast of Spain. According to official data, the approximate population of Catalonia in 2017 was 7.5 million inhabitants, of which 63.9% lived in the metropolitan area of Barcelona [34]. The population density in Catalonia in that year was 235.3 inhabitants per km^2^ (1942.3 inhabitants per km^2^ in the metropolitan area). As for the demographic structure, 18.9% of the Catalan population was 65 years old or older and, within that stratum, 57.1% were women.

### 1.3. Purpose and Contribution of the Study

The main aim of this study is to explore how both fixed and modifiable risk factors affect mental well-being in the last stages of life, with a special emphasis on the influence of the level of rurality of the living environment. Evidence is sought to assess whether living in what are typically densely populated urban areas, with an accelerated rhythm of life, reduces mental well-being in the elderly or, on the contrary, the isolation associated with rural areas contributes more significantly to their psychological discomfort. We specifically aim to address two research questions. First, we seek to identify factors related to low mental well-being of the elderly in the study area. And, second, we aim to assess the extent to which the level of rurality of the home municipality is associated with significantly different values on the mental well-being scale, and whether the magnitude of this association depends on the socio-demographic, health and lifestyle characteristics of the individual.

Our study makes a relevant contribution to the existing literature in the field given that, although some determinants of the emotional well-being of the elderly are known [8,10,18], to the best of our knowledge there are no studies that consider the magnitude of the association between the main variables linked to positive mental well-being and the rural or urban characteristics of the living environment.

## 2. Materials and Methods

### 2.1. Data

We used cross-sectional microdata from the Catalan Health Survey (Enquesta de Salut de Catalunya—ESCA) [35]. This is an official survey conducted among the entire Catalan population and has been repeated twice a year since 2010. Data collection employs computer-assisted face-to-face interviews, and the survey provides extensive information on individuals’ health and lifestyles in relation to a wide set of sociodemographic factors. The sample uses a semi-annual random design, with strata based on age, gender and geographical area [36], and is representative of the global population.

The current study is based on the 2015–2017 sample of 2621 individuals (1219 males and 1402 females) aged 65 years and older that answered the questionnaire personally. The database also included information about 326 individuals of the same age interval that were excluded of our study, as they had been interviewed through an informant using an indirect questionnaire that did not ask about mental well-being. In 38.3% of the cases, the original sample unit could not be reached by the interviewer. Following the ESCA standard procedure [35], a substitute identical to the original in terms of their stratification variables was designated.

### 2.2. Measures

#### 2.2.1. Outcome Measure

The shortened version of the Warwick-Edinburgh Mental Well-being Scale (SWEMWBS) [37] was used to assess the mental well-being for our population of individuals aged 65 and over. The original 14-item scale and its shortened 7-item version were developed and validated in the NHS Health Scotland [38]. The Spanish version scale also meets the validity and reliability requirements [39].

The SWEMWBS measures an individual’s mental well-being in the immediate previous two weeks. Questions relate to feeling optimistic about the future, useful, relaxed, dealing with problems well, thinking clearly, feeling close to other people, and being able to make up their own mind about things. Each item is ranked on a 5-point Likert-type scale from “None of the time” to “All of the time”. The addition of all the items results in a global score, with higher scores indicating better levels of mental well-being (range 7 to 35). In line with the suggestion of the panel of experts [38], we applied a conversion table to the global score. Next, the indicator of mental well-being was built as done in previous studies [40,41]: individuals with global scores that were more than one standard deviation below the mean were considered to present low mental well-being, while the rest were deemed to present normal mental well-being.

#### 2.2.2. Risk Factors

We considered several risk factors that might potentially influence the mental well-being of older people:

Demographic factors: Age groups (65–74, 75–84, 85+), gender, household size (living alone or living with other household members), and educational attainment (low—less than high school vs. middle/high—upper secondary or tertiary).

Economic status: In the absence of reliable data on income, a proxy variable included in the survey was used, namely whether a household makes ends meet at the end of the month with great difficulty, with some difficulty, or easily.

Self-perceived health: Respondents give an overall subjective assessment of their health answering the question: “How is your health in general?”. Responses were grouped into three categories: good (excellent, very good or good), fair or bad.

Physical health burden: Respondents were asked: “Do you have or has a doctor ever told you that you have any of the following conditions (…)?”, followed by a list of 32 chronic medical disorders. We created an indicator for the presence of some selected physical conditions that are usually associated with quality-of-life loss or psychological distress: diabetes, anemia, arthrosis, arthritis, rheumatism, stroke, heart attack or other heart diseases, malignant tumors or Parkinson’s disease [42,43].

Functional limitations and dependence: Level of sensory loss (none, one limitation, or two or more limitations with regard to hearing, seeing, speaking, and writing or reading); presence of limitations for activities of daily living (ADL) (without limitations or slightly limited vs. severely limited); and need for help or company in carrying out ADL (never vs. occasionally or regularly).

Social support: An indicator of low or normal social support was created using the 11-item version of the Duke Social Support Index (DSSI) [44] included in the ESCA questionnaire. It comprises two dimensions: social interactions (frequency of social contact) and subjective support (satisfaction with emotional support provided). The item response options are on a 5-point Likert scale ranging from 1 (much less than I would like) to 5 (as much as I would like). Social support was considered normal if the 11-item global score is >32; otherwise it was considered low.

Family burden: The respondent performs informal care tasks for a disabled person or a person over the age of 75.

Physical activity: The ESCA provides the International Physical Activity Questionnaire, which classifies physical activity as low, moderate or vigorous [45]. The questionnaire provides internationally comparable results. The classification is based on the time the participant has spent being physically active in the last 7 days, taking into account the intensity of this physical activity [46].

Sleep hours: more than 8 h per day, between 6 and 8 h, and less than 6 h. According to the National Sleep Foundation, the recommended number of hours of sleep for health and well-being at older ages are between 7 and 8 per day, though some individuals may make do with fewer [47].

#### 2.2.3. Territorial Factor

The municipalities included in the sample were classified as either rural, semi-rural or urban. Catalonia is divided into 42 districts and 947 municipalities. A predominantly urban district is one in which less than 15% of its municipalities have a population density below 150 inhabitants/km^2^; for semi-rural districts that percentage is between 15 and 50%; finally, in the case of predominantly rural districts, the percentage exceeds 50% [48]. Initially, each municipality in our database was assigned the level of rurality of its corresponding district. Then we adjusted it based on criteria provided by the Spanish National Institute of Statistics [49], given the need to identify more accurately some individual municipalities in relation to the typology of their district. Thus, a semi-rural typology was assigned to municipalities in rural districts with a population greater than 10,000 inhabitants and to municipalities in urban districts with a population of less than 10,000 inhabitants; similarly, all municipalities with less than 2000 inhabitants were considered rural. According to these new criteria, in 2017, 6.6% of the Catalan population lived in rural municipalities; 26.4% in semi-rural municipalities; and 67.0% in urban municipalities. The surface area of the rural, semi-rural and urban municipalities corresponds to 73.3, 21.4 and 5.3%, respectively, of the total Catalan territory.

### 2.3. Analytical Strategy

To address our first research question, we divided the individuals in our sample according to the rural, semi-rural or urban typology of their home municipality. We then described the sociodemographic, health and lifestyle characteristics of each of these three groups (Table 1). Next, we ran a logistic regression model for normal vs. low mental well-being according to the SWEMWBS score recorded (Table 2). The odds-ratios significance was tested at the 1%, 5% and 10% significance levels. Several risk factors, including the level of rurality of the municipality of residence, were used as regressors. We tested for heteroscedasticity and multicollinearity and obtained negative results.

To address our second research question, a comparison of the SWEMWBS scores by risk factor and level of rurality was conducted (Table 3). The asymmetric nature of the SWEMWBS distributions did not allow an analysis of variance to be performed. Instead, we used non-parametric methods to test if one of the distributions did not have the same shape (and, possibly, median) as the rest, without requiring the normality hypothesis of the distributions to be met [50]. Significance levels of 1% and 5% were used. We performed the Kruskal-Wallis (KW) H-test when comparing the score distributions for the different categories of a risk factor (e.g., the 65–74, 75–84 and 85+ age groups). We then used the same test when simultaneously comparing the three groups of different levels of rurality for a subsample of individuals (e.g., those aged 65–74). Finally, to identify significant differences between the score distributions of a subsample for each pair of territory typologies, the Mann-Whitney (MW) U-test with Bonferroni correction was used (e.g., scores for individuals aged 65–74 for rural vs. semi-rural home municipality).

Sampling weights provided by the survey’s panel of experts were used in the analyses in Table 2 and Table 3 to correct for age and gender deviations when comparing the sample structure and that of the Catalan population as a whole. All statistical analyses were performed using IBM^®^ SPSS^®^ 25 (IBM, Armonk, NY, USA).

## 3. Results

Table 1 shows the sample characteristics by the level of rurality of the home municipality. The more urban the area of residence, the lower is the average age of the sample, and the higher the proportion of women and respondents with middle or high education. However, the percentage of persons living alone was very similar (around 22%) regardless of the level of rurality. Rural residents seemed to make ends meet more easily than the rest (72.8% declared no difficulty vs. 61.1% for semi-rural and 62.4% for urban areas).

As for self-perceived health, respondents resident in semi-rural areas reported feeling healthier, despite their presenting the highest morbidity level (74.0% declared themselves to be suffering from some of the selected diseases vs. 70.4% of those living in urban areas) and the highest percentage of individuals facing serious limitations for ADL. Rural residents enjoyed the greatest social support, which was considered normal in 97.7% of cases, two points above the corresponding values for semi-rural and urban areas. As for lifestyle, the level of physical activity in urban areas was higher than in the other two areas (65.3% presented moderate or vigorous physical activity vs. around 60% in the semi-rural and rural areas). People living in urban areas dedicated the fewest hours to sleeping: only 15.8% reported sleeping more than 8 h per day vs. 20.8% in semi-urban and 26.1% in rural areas. The rates of respondents being a caregiver for a disabled person or individual aged 75+ or needing help with ADL themselves were similar across the three municipality types. Finally, our sample results show that a higher level of rurality is associated with a better level of mental well-being. Thus, while in urban areas 21.4% of the sample individuals had deficient scores, this percentage was 17.4% for semi-rural and 12.9% for rural residents.

The logistic regression model helps identify some of the factors linked to low mental well-being measured through the SWEMWBS scores (Table 2). According to this multivariate analysis, there was no significant evidence that being female or being older were features associated with a higher risk of poor mental well-being. Conversely, individuals that lived alone (OR = 1.36, 95% CI = (1.00; 1.84), *p* = 0.049), had low primary education (OR = 1.74, 95% CI = (1.33; 2.27), *p* < 0.001), or experienced great or some difficulty to reach the end of the month on their income (OR = 1.69, 95% CI = (1.20; 2.39), *p* = 0.003; OR = 1.49, 95% CI = (1.12; 1.97), *p* = 0.006, respectively) were likely to have a low level of mental well-being.

Variables relating to health status, personal autonomy and social support seemed to be strongly associated with mental well-being. Individuals with poor or fair self-perceived health presented a greater risk of low mental well-being than those reporting good health (OR = 4.19, 95% CI = (2.79; 6.29), *p* < 0.001; OR = 2.38, 95% CI = (1.80; 3.14), *p* < 0.001, respectively). Similarly, those suffering from one of the selected physical diseases (OR = 1.52, 95% CI = (1.06; 2.18), *p* = 0.022), with one, or two or more, sensory limitations (OR = 1.48, 95% CI = (1.03; 2.14), *p* = 0.034; OR = 2.69, 95% CI = (1.61; 4.47), *p* < 0.001, respectively), facing severe limitations for ADL (OR = 1.97, 95% CI = (1.29; 3.00), *p* = 0.002), a lack of personal autonomy (OR = 3.23, 95% CI = (2.44; 4.28), *p* < 0.001), low social support (OR = 3.86, 95% CI = (2.34; 6.37), *p* < 0.001) or being informal caregivers of a disabled person or individual aged 75+ (OR = 1.37, 95% CI = (0.99; 1.91), *p* = 0.058) had a greater probability of low mental well-being.

According to our results, leading a physically active life and getting enough sleep are positively correlated with normal mental well-being. Specifically, those with low or moderate levels of physical activity had a higher probability of poor mental well-being (OR = 2.84, 95% CI = (1.51; 5.37), *p* = 0.001; OR = 2.17, 95% CI = (1.16; 4.08), *p* = 0.015, respectively) than older persons that engaged in vigorous physical activity. Likewise, lack of sleep is linked to worse mental well-being: those who slept less than 6 h a day, or even between 6 and 8 h, were at a higher risk of poor mental well-being (OR = 2.12, 95% CI = (1.39; 3.23), *p* = 0.001; OR = 1.43, 95% CI = (1.03; 1.98), *p* = 0.032, respectively) than those sleeping more than 8 h.

Finally, individuals living in urban areas were twice as likely to experience mental distress than those living in rural areas (OR = 2.00, 95% CI = (1.41; 2.83), *p* < 0.001). This effect is somewhat lower among those living in semi-rural areas, although it is still significant at a 10% level (OR = 1.44, 95% CI = (0.99; 2.08), *p* = 0.053).

To address the second research question we calculated the median SWEMWBS scores by level of rurality (Table 3). The vast majority of the results show statistically significant differences between the distribution shapes when we compare the categories of each risk factor. Different levels of self-perceived health are connected with especially relevant disparities in the SWEMWBS distributions, which are transferred to the values of the medians: thus, there is a difference of 4.6 points between the median SWEMWBS scores for good (27.0) and poor health (22.4) in rural areas (KW = 40.8, *p* < 0.001, when comparing the distribution shapes). This difference is even greater in semi-rural (9.3 points, KW = 162.3, *p* < 0.001) and urban communities (6.0 points, KW = 172.2, *p* < 0.001). Significant reductions in the median mental well-being scores are also observed when there were severe limitations for, or a need for help with, ADL, above all in semi-rural areas, with a 7.7- and 6.6-point difference, respectively, between those who did not suffer severe limitations or did not need help and those who did (KW = 86.5, *p* < 0.001; KW = 138.8, *p* < 0.001, respectively). These differences are slightly lower for those in urban areas (5.7 points, KW = 75.3, *p* < 0.001; 5.3 points, KW = 195.6, *p* < 0.001, respectively for both factors), and even smaller for those in rural communities (4.5 points, KW = 21.8, *p* < 0.001; 3.8 points, KW = 64.7, *p* < 0.001, respectively). In contrast, social support seems to correlate with mental well-being to a greater extent in semi-rural and rural areas (6.3-point difference, KW = 25.5, *p* < 0.001; 5.3-point difference, KW = 4.9, *p* = 0.028, respectively, when comparing the SWEMWBS scores for normal and low support).

The overall SWEMWBS score distributions for rural and semi-rural residents show no significant differences (MW = −0.1, *p* = 0.960), while lower scores of mental well-being were found for urban residents (MW = −4.9, *p* < 0.001; MW = −5.4, *p* < 0.001, for rural and semi-rural areas, respectively). When the three groups are compared jointly, most differences are statistically significant as well.

Pairwise comparisons by level of rurality reveal few differences between rural and semi-rural distributions, limited in this instance to fair or good health (MW = −2.5, *p* = 0.011; MW = −2.4, *p* = 0.015, respectively), and severe limitations for undertaking ADL (MW = −2.5, *p* = 0.011), indicating better mental well-being for rural residents. Nevertheless, a comparison between rural/semi-rural residents and those living in urban communities reveals almost all peer differences to be significant.

Without exception, the MW z-statistic presented negative values, indicating that urban residents present a poorer mental well-being. An inspection of the median scores shows that the differences between rural and urban residents were especially large and highly significant (*p* < 0.001 for the MW test with Bonferroni correction, unless otherwise stated) for individuals aged 65–74 (MW = −4.4); living in company (MW = −3.9); with primary or no education (MW = −5.4); making ends meet with some difficulty (MW = −4.4); with fair self-perceived health (MW = −3.6); suffering from some of the selected diseases (MW = −4.8); with only one sensory limitation (MW = −3.2, *p* = 0.001); needing help with ADL (MW = −3.3, *p* = 0.001); with normal social support (MW = −4.7); not being caregivers for a disabled person or individual aged 75+ (MW = −5.1); and sleeping less than 6 h a day (MW = −2.9, *p* = 0.004). In addition, residents of rural areas show much higher mental well-being scores than those of urban environments for all levels of physical activity.

Comparisons between those living in semi-rural or urban communities yield similar results in the case of sociodemographic variables but present a number of differences in the case of health, social support and lifestyle factors. Differences in median well-being scores were particularly high, with significant MW tests (*p* < 0.001, unless otherwise stated) for residents reporting good health (MW = −6.0); no physical diseases (MW = −3.6); no sensory loss (MW = −5.4); no severe limitations for ADL (MW = −6.1); no need for help with ADL (MW = −5.8); normal social support (MW = −5.8); vigorous physical activity (MW = −3.4, *p* = 0.001), and sleeping between 6 and 8 h a day (MW = −5.0).

## 4. Discussion

Subjective and objective poor health results in emotional distress, often causing anxiety and depression in the elderly. Likewise, sensory or functional limitations, typically associated with a need for help in undertaking ADL, carry an emotional burden for the elderly, who see their personal autonomy undermined. This burden is further aggravated if the individual is herself an informal caregiver for disabled persons or individuals aged over 75. If this care is provided in rural areas, where access to shops and services is difficult, the caregiver may be at an increased risk of poor mental health [51]. Living alone and having low social support are two further risk factors that should be considered, reinforcing what is already known about the importance of interacting with people in all stages of life, especially at older ages [52]. Finally, physical activity and sleeping a sufficient number of hours appear to be associated with less emotional distress no matter the level of rurality.

In terms of mental well-being, our findings reveal that those who enjoy a better status when living in a rural environment are men and women under the age of 85, with no serious problems of getting to the end of the month on their income, with fair or good self-perceived health, suffering physical comorbidities but with one sensory limitation at the most, with no severe problems performing ADL, and with normal social support. They are also people who do not have to provide care for a disabled individual or someone over the age of 75, and who get by with little or a regular amount of sleep. Thus, we can conclude that although rural dwellers may suffer some physical conditions or face mild limitations, being optimistic about their own health, obtaining social support and living in the countryside may exert a positive influence and bolster their mental health.

Similarly, the differential benefits of living in a semi-rural environment as opposed to an urban area seem to be relevant for men and women below the age of 85, not living alone, of any educational level, having good self-perceived health, no sensory loss, no severe limitations for ADL, no need for help with ADL, normal social support, no dependents to take care of, and sleeping 6 to 8 h/day.

As people grow older they experience greater physical and mental health problems which need addressing. Health officials face the challenge of ensuring that the elderly can enjoy good quality of life during the last stages of their life. Our findings strengthen the belief that good social welfare policies are crucial. Policy makers should actively seek to plan specific service provisions for different geographical areas presenting different demographic patterns. Among key policy considerations, the prevention of loneliness, especially in urban areas, has to be prioritized along with policies that allow older people to ‘age in place’ and which provide easy access both to physical and mental healthcare and social care. For instance, a well-developed transportation system is essential to prevent social exclusion [53]. In short, social policies should seek to address not solely health issues but well-being in all its facets, the latter being especially crucial for urban residents.

While this paper makes a relevant contribution to the literature, certain limitations inherent to the study may arguably have affected our results. Given the fact that the ESCA does not target individuals in nursing homes, generalizing the findings to the entire 65+ population is not possible. Moreover, caution is required when considering elderly populations elsewhere, because of different levels of economic development and the operational definition of rurality that has been used in this study. Additionally, all the scales employed were obtained from surveys. This may lead to the introduction of some bias in the results as participants may well under- or overestimate their subjective characteristics due to an incorrect assessment in the presence of an interviewer. Further research addressing these concerns is needed to add to our understanding of the relationship between mental well-being across territories and individuals’ lives. Conducting quantitative and qualitative studies that add depth to our findings about rurality would allow us to further explore the mechanisms that link rurality and the well-being of the elderly.

## 5. Conclusions

The aim of this paper was to examine the chief factors associated with mental well-being at older ages, with a particular focus on the level of rurality of the municipality in which the individual was resident. Our findings identify various fixed and modifiable risk factors that are linked to mental well-being and which point to substantial differences depending on the level of rurality of the living environment.

A negative association between low mental well-being and being female or being older is evident in both rural and urban environments; however, the level of mental well-being is mainly captured by existing differences in health status and personal autonomy [54]. Our findings reveal that the mental risk is aggravated in urban areas. It is worth noting that while an individual’s socioeconomic and educational levels can hardly be modified in their old age, they can be influenced during earlier stages of life through adequate policies that target specific groups of individuals.

The key contribution made by our paper is to show that the level of rurality of an environment is clearly associated with the level of mental well-being of its residents. Our findings reveal that the higher the level of rurality of the municipality of residence, the better the level of mental well-being of those residents there. Incentivizing older people to live in rural environments could lead to greater well-being in later life; this positive outcome should be tested in appropriate prospective studies.

## Figures and Tables

**Table 1:** Sample characteristics by level of rurality of home municipality. Individuals aged 65+.

		Level of Rurality			
	n	Total	Rural (n = 527)	Semi-Rural (n = 872)	Urban (n = 1222)
*Age in years, mean (SD)*	2621	76.1 (7,7)	76.6 (8.0)	76.3 (7.9)	75.7 (7.5)
*Age groups*	2621				
65–74		47.6	44.8	46.6	49.6
75–84		34.7	35.9	34.9	34.1
85+		17.7	19.4	18.6	16.3
*Gender*	2621				
Male		46.5	48.0	47.9	44.8
Female		53.5	52.0	52.1	55.2
*Household size*	2621				
Living alone		22.1	22.2	22.1	22.1
More than one member		77.9	77.8	77.9	77.9
*Level of education*	2620				
Middle or high		42.9	37.8	41.4	46.3
Low		57.1	62.2	58.6	53.7
*How do they make ends meet?*	2613				
With great difficulty		11.7	8.9	12.1	12.7
With some difficulty		24.2	18.3	26.8	24.9
With ease		64.1	72.8	61.1	62.4
*Self-perceived health*	2620				
Poor		8.5	8.0	7.2	9.6
Fair		32.5	32.3	31.0	33.7
Good		59.0	59.8	61.8	56.8
*Physical diseases*	2621				
Yes		72.0	72.7	74.0	70.4
No		28.0	27.3	26.0	29.6
*Sensory loss*	2621				
2 or more limitations		3.7	3.8	4.1	3.4
Only one limitation		8.9	10.6	7.6	9.1
Without limitations		87.4	85.6	88.3	87.5
*Severe limitation for ADL*	2621				
Yes		6.7	5.9	7.3	6.6
No		93.3	94.1	92.7	93.4
*Needs help with ADL*	2621				
Yes		21.8	20.1	22.0	22.4
No		78.2	79.9	78.0	77.6
*Social support*	2608				
Low		3.9	2.3	4.3	4.3
Normal		96.1	97.7	95.7	95.7
*Caregiver of disabled/75+*	2621				
Yes		18.4	17.1	19.5	18.2
No		81.6	82.9	80.5	81.8
*Physical activity*	2621				
Low		37.5	39.1	40.6	34.7
Moderate		53.3	51.6	50.2	56.3
Vigorous		9.1	9.3	9.2	9.0
*Sleeping hours*	2615				
Less than 6 h/day		11.4	9.5	11.8	11.8
6 to 8 h/day		69.1	64.4	67.4	72.4
More than 8 h/day		19.5	26.1	20.8	15.8
*Mental well-being*	2621				
Low		18.4	12.9	17.4	21.4
Normal		81.6	87.1	82.6	78.6

Source: Catalan Health Survey (ESCA), 2015–2017. Note: Numbers are percentages (%) unless otherwise stated. n = sample size. SD: standard deviation. ADL: Activities of daily living.

**Table 2:** Logistic regression model for the SWEMWBS indicator (1 = low; 0 = normal).

	Odds-ratio		95% CI
*Age groups* (ref. 65–74)			
75–84	0.92		(0.72; 1.19)
85+	1.18		(0.88; 1.58)
*Gender* (ref. Male)			
Female	1.20		(0.85; 1.71)
*Household size* (ref. More than one member)			
Living alone	1.36	**	(1.00; 1.84)
*Level of education* (ref. Middle or high)			
Low	1.74	***	(1.33; 2.27)
*How do they make ends meet?* (ref. With ease)			
With great difficulty	1.69	***	(1.20; 2.39)
With some difficulty	1.49	***	(1.12; 1.97)
*Self-perceived health* (ref. Good)			
Poor	4.19	***	(2.79; 6.29)
Fair	2.38	***	(1.80; 3.14)
*Physical diseases* (ref. No)			
Yes	1.52	**	(1.06; 2.18)
*Sensory loss* (ref. Without limitations)			
2 or more limitations	2.69	***	(1.61; 4.47)
Only one limitation	1.48	**	(1.03; 2.14)
*Severe limitation for ADL* (ref. No)			
Yes	1.97	***	(1.29; 3.00)
*Needs help with ADL* (ref. No)			
Yes	3.23	***	(2.44; 4.28)
*Social support* (ref. Normal)			
Low	3.86	***	(2.34; 6.37)
*Caregiver of disabled/75+* (ref. No)			
Yes	1.37	*	(0.99; 1.91)
*Physical activity* (ref. Vigorous)			
Low	2.84	***	(1.51; 5.37)
Moderate	2.17	**	(1.16; 4.08)
*Sleeping hours* (ref. More than 8 h/day)			
Less than 6 h/day	2.12	***	(1.39; 3.23)
6 to 8 h/day	1.43	**	(1.03; 1.98)
*Level of rurality* (ref. Rural)			
Semi-rural	1.44	*	(0.99; 2.08)
Urban	2.00	***	(1.41; 2.83)

Source: Catalan Health Survey (ESCA), 2015–2017. Sample size (after listwise deletion): n = 2581. *** *p* < 0.01, ** *p* < 0.05, * *p* < 0.10. SWEMWBS: Warwick Edinburgh Mental Well-Being Scale, short version. CI: Confidence interval. Ref.: Reference category. ADL: Activities of daily living.

**Table 3:** SWEMWBS scores by risk factor and level of rurality.

	Rural		Semi-rural		Urban		3-Group Comparison	Multiple Comparisons		
	Median Score	KW	Median Score	KW	Median Score	KW	KW	R vs. SR (MW)	R vs. U (MW)	SR vs. U (MW)
*TOTAL*	26.0	-	26.0	-	25.0	-	39.5 **	−0.1	−4.9 ^b^	−5.4 ^b^
*Age groups*										
65–74	27.0	28.3 **	27.0	21.4 **	25.0	35.3 **	26.8 **	−0.7	−4.4 ^b^	−4.0 ^b^
75–84	25.0		25.0		25.0		13.9 **	−0.5	−2.6 ^a^	−3.4 ^b^
85+	25.0		25.0		23.2		7.0 *	−0.1	−2.3	−2.1
*Gender*										
Male	27.0	4.4 *	27.0	9.2 **	26.0	21.0 **	14.1 **	−0.4	−2.6 ^a^	−3.5 ^b^
Female	26.0		25.0		25.0		24.4 **	−0.3	−4.2 ^b^	−4.0 ^b^
*Household size*										
Living alone	25.0	0.9	25.0	9.6 **	25.0	9.3 **	10.8 **	−1.3	−3.2 ^b^	−2.0
More than one member	26.0		27.0		25.0		31.6 **	−0.7	−3.9 ^b^	−5.1 ^b^
*Level of education*										
Middle or high	27.0	7.4 **	27.0	15.6 **	26.0	58.9 **	10.9 **	−0.4	−2.2	−3.0 ^b^
Low	25.0		25.0		24.1		38.4 **	−0.6	−5.4 ^b^	−5.0 ^b^
*How do they make ends meet?*										
With great difficulty	23.2	8.0 *	23.2	37.6 **	22.4	100.8 **	5.7	−0.9	−2.2	−1.6
With some difficulty	26.0		25.0		24.1		28.7 **	−0.7	−4.4 ^b^	−4.4 ^b^
With ease	26.0		27.0		26.0		13.3 **	−1.6	−1.5	−3.6 ^b^
*Self-perceived health*										
Poor	22.4	40.8 **	20.0	162.3 **	20.0	172.2 **	4.9	−2.0	−2.0	−0.1
Fair	26.0		24.1		24.1		13.3 **	−2.5 ^a^	−3.6 ^b^	−1.1
Good	27.0		29.3		26.0		36.1 **	−2.4 ^a^	−2.6 ^a^	−6.0 ^b^
*Physical diseases*										
Yes	26.0	14.9 **	25.0	30.0 **	25.0	58.8 **	34.0 **	−0.3	−4.8 ^b^	−4.9 ^b^
No	27.0		28.1		27.0		13.8 **	−0.9	−2.1	−3.6 ^b^
*Sensory loss*										
2 or more limitations	20.7	17.7 **	22.4	30.0 **	20.0	49.7 **	2.0	−0.2	−0.9	−1.4
Only one limitation	26.0		23.2		22.4		10.4 **	−2.1	−3.2 ^b^	−0.9
Without limitations	26.0		27.0		25.0		35.4 **	−0.7	−4.2 ^b^	−5.4 ^b^
*Severe limitation for ADL*										
Yes	21.5	21.8 **	19.3	86.5 **	19.3	75.3 **	6.3 *	−2.5 ^a^	−2.0	−0.7
No	26.0		27.0		25.0		44.2 **	−0.9	−4.6 ^b^	−6.1 ^b^
*Needs help with ADL*										
Yes	23.2	64.7 **	21.5	138.8 **	20.7	195.6 **	10.4 **	−1.7	−3.3 ^b^	−1.6
No	27.0		28.1		26.0		38.0 **	−1.1	−4.0 ^b^	−5.8 ^b^
*Social support*										
Low	20.7	4.9 *	20.7	25.5 **	21.5	14.7 **	0.7	−0.7	−0.2	−0.7
Normal	26.0		27.0		25.0		42.0 **	−0.5	−4.7 ^b^	−5.8 ^b^
*Caregiver of disabled/75+*										
Yes	25.0	9.0 **	25.0	8.7 **	25.0	1.0	0.9	−0.4	−0.3	−0.9
No	26.0		27.0		25.0		42.5 **	−0.1	−5.1 ^b^	−5.6 ^b^
*Physical activity*										
Low	25.0	27.1 **	25.0	62.7 **	23.2	86.2 **	24.8 **	−1.3	−4.6 ^b^	−3.6 ^b^
Moderate	26.0		27.0		25.0		20.4 **	−1.3	−2.5 ^a^	−4.7 ^b^
Vigorous	29.3		30.7		27.0		14.6 **	−0.5	−2.9 ^a^	−3.4 ^b^
*Sleeping hours*										
Less than 6 h/day	27.0	2.6	24.1	18.0 **	23.2	22.1 **	8.1 *	−1.7	−2.9 ^a^	−1.1
6 to 8 h/day	26.0		27.0		25.0		31.4 **	−0.3	−4.0 ^b^	−5.0 ^b^
More than 8 h/day	26.0		27.0		25.0		4.6	−0.9	−1.1	−2.1

Note: Kruskal-Wallis (KW) non-parametric H tests used for comparisons between the distribution shapes of the variable categories and between rurality levels due to skewed distributions. * *p* < 0.05, ** *p* < 0.01. Multiple comparisons conducted with Mann-Whitney (MW) U test (z-statistic reported) with a Bonferroni correction to the alpha level of: ^a^ 0.05/3 (0.017); ^b^ 0.01/3 (0.003). SWEMWBS: Warwick Edinburgh Mental Well-Being Scale, short version. R = rural; SR = semi-rural; U = urban. ADL: Activities of daily living.
